# A positive linear association between uric acid and the risk of gestational diabetes mellitus: a single-center retrospective study

**DOI:** 10.3389/fendo.2026.1685036

**Published:** 2026-02-10

**Authors:** Mengfan Zhang, Chunbo Li, Xiaoli Zhao, Mengchun Li, Zhaohua Tian

**Affiliations:** 1 Department of Obstetrics and Gynecology, The Fifth Clinical Medical College of Henan University of Chinese Medicine (Zhengzhou People’s Hospital), Zhengzhou, China; 2 Department of General Surgery, The Fifth Clinical Medical College of Henan University of Chinese Medicine (Zhengzhou People’s Hospital), Zhengzhou, China; 3 Department of Pediatric Development and Behavior, The Third Affiliated Hospital of Zhengzhou University, Zhengzhou, China

**Keywords:** gestational diabetes mellitus, pregnancy, restricted cubic spline, retrospective cohort study, uric acid

## Abstract

**Objective:**

This study aimed to examine the association between maternal uric acid (UA) concentrations measured prior to 24 weeks of pregnancy and the risk of gestational diabetes mellitus (GDM) between 24 and 28 weeks.

**Methods:**

In this retrospective cohort conducted at a single institution, 847 expectant mothers receiving prenatal services at Zhengzhou People’s Hospital from August to December 2024 were enrolled. All participants had their UA levels measured prior to 24 gestational weeks, followed by a 75 g oral glucose tolerance assessment conducted between weeks 24 and 28. Multivariable logistic regression analysis was performed to evaluate the association between UA levels and the risk of GDM, supplemented by subgroup analysis, sensitivity analysis, and analytical procedures including receiver operating characteristic (ROC) curve evaluation and modeling with restricted cubic spline (RCS).

**Results:**

Among 847 enrolled pregnancies, the proportion with GDM was 21.1% (n = 179). Multivariate logistic regression showed that each 1 µmol/L or each one-standard-deviation elevation in serum UA corresponded to a markedly greater risk of GDM (OR: 1.005, 95% CI: 1.001–1.009; OR: 1.274, 95% CI: 1.062–1.528, respectively). In comparison to the lowest UA tertile (T1), T2 and T3 exhibited elevated risk for GDM, with ORs of 1.976 (95% CI: 1.203–3.247) and 2.468 (95% CI: 1.520–4.007), respectively. Subgroup analyses revealed that in women aged > 30, with gravidity < 2, parity < 2, or body mass index before conception below 24 kg/m^2^, both continuous and categorical UA demonstrated a clear and statistically meaningful association with GDM. Sensitivity analyses supported consistent results, with both median- and quartile-based groupings showing a significant association between higher UA levels and greater GDM risk (P < 0.05). ROC curve analysis suggested that serum UA provided limited yet statistically significant discriminative information for GDM (AUC = 0.623). RCS modeling revealed a notable positive linear association between UA concentrations and the risk of GDM (P for nonlinearity = 0.140).

**Conclusion:**

Higher maternal serum UA measurements taken prior to 24 gestational weeks were strongly linked to GDM risk during weeks 24–28.

## 1 Introduction

Gestational diabetes mellitus (GDM), defined as glucose intolerance initially detected in pregnancy, is increasing in prevalence worldwide and presents a major public health concern affecting the health of both mothers and their offspring (1). GDM not only elevates the likelihood of hypertensive disorders of pregnancy, surgical delivery, and the onset of type 2 diabetes after childbirth but also poses significant threats to fetal outcomes, including macrosomia, preterm birth, neonatal hypoglycemia, and respiratory distress syndrome (1–3). Therefore, timely detection and management of key risk factors for GDM are essential to enhance outcomes for both mothers and newborns.

Recently, the importance of metabolic indicators in predicting GDM at an early stage has gained growing research interest (4). Uric acid (UA), the end metabolite of purine breakdown, has long been linked to health issues including gout, high blood pressure, heart diseases, and increased mortality (5–7). However, increasing research indicates that UA could be involved in the onset of insulin resistance (8). The physiological fluctuations of uric acid during pregnancy are influenced by various factors, including changes in glomerular filtration rate, placental hormone secretion, and maternal metabolic alterations (9, 10). Several studies have shown that elevated UA levels before pregnancy, during early pregnancy, and in mid-pregnancy are linked to the emergence of GDM during the later phases of gestation (11–14). Nevertheless, the association between UA and GDM is still debated, as research results vary among studies and the exact mechanisms remain unclear (11, 15). Moreover, variations in genetics, living habits, diet, and geographic conditions may lead pregnant women in specific regions of China to exhibit distinct metabolic profiles and unique predispositions to GDM (16). Therefore, comprehensive investigation into the association between UA concentrations and GDM among Chinese individuals is of paramount importance, as it would not only help to more accurately identify high-risk individuals but also provide scientific evidence for developing more targeted intervention strategies.

In light of this context, this study conducted a retrospective investigation examining the association between UA concentrations and GDM risk within a single-center cohort. The aim was to assess whether UA could function as an early biochemical indicator for GDM, offering clinical value for early risk identification and supporting the broader integration of metabolic markers into perinatal care practices, with the ultimate goal of promoting better outcomes for both mothers and their babies.

## 2 Methods

### 2.1 Study design and population

The research was designed as a retrospective cohort study and carried out at the Zhengzhou People’s Hospital. A total of 847 pregnant participants, all of whom obtained standard prenatal care and gave birth at the hospital from August to December 2024, were enrolled according to the preset eligibility criteria.

Participants met the subsequent eligibility requirements: (1) aged 18 years or older; (2) established prenatal records at the study hospital in early pregnancy and regular follow-up; (3) singleton pregnancy; (4) complete serum UA data available before 24 weeks of gestation; and (5) completion of a 75-gram oral glucose tolerance test (OGTT) conducted during gestational weeks 24–28. The exclusion criteria included: (1) pre-existing diabetes or other severe endocrine or metabolic disorders prior to conception; (2) pre-existing disorders influencing UA metabolism, including chronic kidney disease, gout, and systemic lupus erythematosus; (3) coexisting major pregnancy complications, including severe preeclampsia; and (4) pregnant women with missing values in any clinical variable. The study received ethical clearance from the Zhengzhou People’s Hospital Ethics Committee and adhered to the principles of the Declaration of Helsinki. Given the retrospective nature of the study and the anonymization of all patient data during analysis, informed consent from participants was waived.

### 2.2 Gestational diabetes mellitus: evaluation and categorization

All participants completed a standard 75 g OGTT between the 24th and 28th weeks of gestation as part of routine prenatal evaluation. Following the diagnostic guidelines of the International Association of Diabetes and Pregnancy Study Groups (IADPSG), endorsed by the Chinese Medical Association, GDM was identified when at least one of the following values was reached: 1-hour post-load glucose ≥ 10.0 mmol/L, 2-hour post-load glucose ≥ 8.5 mmol/L, or fasting plasma glucose (FPG) ≥ 5.1 mmol/L (17). According to OGTT outcomes, 847 pregnant women were enrolled, including 179 cases of GDM and 668 classified as non-GDM.

### 2.3 Data collection and variable measurement

This study obtained clinical and laboratory information from the electronic medical records of Zhengzhou People’s Hospital. All samples and measurements were obtained during routine prenatal check-ups conducted before 24 weeks of gestation (i.e., from early pregnancy to the early second trimester). Fasting blood specimens were obtained and analyzed in the hospital laboratory according to standardized procedures. Laboratory evaluations included biochemical markers such as UA, FPG, glycated hemoglobin (HbA1c), serum creatinine, blood urea nitrogen (BUN), total bilirubin, and albumin; hematological indices like hemoglobin, white blood cell count (WBC), and platelet count; lipid-related parameters including total cholesterol, triglycerides, high-density lipoprotein cholesterol (HDL-C), and low-density lipoprotein cholesterol (LDL-C); as well as liver enzymes alanine aminotransferase (ALT) and aspartate aminotransferase (AST), along with fibrinogen levels. Demographic and clinical data were also obtained, covering factors such as gravidity, parity, maternal age, pre-pregnancy body mass index (BMI), history of assisted reproduction, and measurements of systolic (SBP) and diastolic blood pressure (DBP).

Maternal serum UA served as the main exposure variable and was assessed both continuously and in categorical groupings. For continuous analysis, UA was included in its original form (µmol/L) as well as a standardized z-score, calculated as: standardized UA = (individual UA – mean UA)/standard deviation (SD). For categorical analysis, participants were grouped using multiple methods. First, they were divided into tertiles: T1 (UA ≤ 221 µmol/L, n = 283), T2 (221 < UA ≤ 258 µmol/L, n = 283), and T3 (UA > 258 µmol/L, n = 281). Second, using the median UA value of 237 µmol/L, they were classified into a low-UA group (≤ 237 µmol/L, n = 425) and a high-UA group (> 237 µmol/L, n = 422). Third, a quartile-based grouping was applied, dividing participants into Q1 (UA ≤ 213 µmol/L, n = 218), Q2 (213 < UA ≤ 237 µmol/L, n = 207), Q3 (237 < UA ≤ 274 µmol/L, n = 215), and Q4 (UA > 274 µmol/L, n = 207).

### 2.4 Statistical analysis

Statistical analyses were carried out with R (v4.4.3; R Foundation for Statistical Computing, Vienna, Austria) and SPSS (v26.0; IBM Corp., Armonk, NY, USA). Maternal demographic and clinical variables were summarized by serum UA tertiles. The Shapiro–Wilk test indicated non-normality for all continuous variables, which were therefore reported as medians with interquartile ranges. Comparisons between groups used the Kruskal–Wallis test for continuous data and the chi-square test for categorical data.

The association between serum UA levels and GDM risk was examined using multivariable logistic regression, with predictors showing P < 0.05 in univariate analysis entered into the models. Odds ratios (ORs) with 95% confidence intervals (CIs) were derived. Four regression frameworks were applied: Model 1 contained no covariate adjustment; Model 2 controlled for age, assisted reproduction, gravidity, parity, and pre-pregnancy BMI; Model 3 was constructed as a sensitivity analysis model excluding FPG and HbA1c to address potential overadjustment, and was additionally adjusted for WBC, hemoglobin, triglycerides, AST, BUN, and fibrinogen; Model 4 was defined as the fully adjusted model, which further included FPG and HbA1c on the basis of Model 3 to control for baseline glycemic status. UA was assessed in multiple formats, including continuous measurement, standardized z-scores, tertiles (T1 as reference), median-based categories (low UA as reference), and quartiles (Q1 as reference). Sensitivity analyses were applied to test the consistency of results, using logistic regression with UA groups defined by both median and quartile cutoffs.

In addition, subgroup analyses were performed by stratifying participants according to maternal age (≤ 30 or > 30 years), gravidity (< 2 or ≥ 2), parity (< 2 or ≥ 2), and BMI before conception (< 24 or ≥ 24 kg/m^2^) to examine whether the observed associations were consistent across varying population strata. In these subgroup models, all covariates from Model 4 were adjusted for except the stratification variable itself, which was excluded from the adjustment to avoid over-controlling for the subgroup-defining factor. The potential of UA to predict GDM was additionally examined using a receiver operating characteristic (ROC) curve, from which the area under the curve (AUC) and its 95% CI were obtained. Additionally, a restricted cubic spline (RCS) regression was applied to examine the nonlinear association between UA levels and the risk of GDM, with four knots placed at the 5th, 35th, 65th, and 95th percentiles of the UA distribution. All analyses were conducted using two-tailed tests, with statistical significance defined as P < 0.05.

## 3 Results

### 3.1 Demographics and clinical features across uric acid tertile groups

As detailed in **Table 1**, a total of 847 pregnant participants were enrolled, with a median age of 32 years, and 179 (21.1%) were diagnosed with GDM. Based on serum UA tertile values, they were classified into T1 (≤ 221 µmol/L), T2 (221–258 µmol/L), and T3 (> 258 µmol/L). Significant variations were found among tertile groups for multiple clinical and metabolic indicators, such as pre-pregnancy BMI, SBP, DBP, triglycerides, ALT, BUN, FPG, HbA1c, and fibrinogen (all P < 0.05). Conversely, no meaningful intergroup differences were observed for maternal age, assisted reproduction, gravidity, parity, WBC, hemoglobin, platelet count, HDL-C, LDL-C, total cholesterol, AST, total bilirubin, albumin, or serum creatinine (all P > 0.05). Notably, GDM prevalence rose steadily with increasing UA levels, from 11.7% in T1 to 21.6% in T2 and 30.2% in T3, showing a statistically significant upward trend (P < 0.001).

**Table 1:** Baseline characteristics by uric acid tertiles.

Variables	Total population	T1	T2	T3	P value
N	847	283	283	281	
Age, years	32.00 (29.00, 35.00)	31.00 (29.00, 35.00)	32.00 (29.00, 35.00)	32.00 (29.00, 35.00)	0.959
Assisted reproduction, n (%)	104 (12.3)	36 (12.7)	27 (9.5)	41 (14.6)	0.181
Gravidity, n (%)					0.228
< 2	420 (49.6)	152 (53.7)	136 (48.1)	132 (47.0)	
≥ 2	427 (50.4)	131 (46.3)	147 (51.9)	149 (53.0)	
Parity, n (%)					0.170
< 2	584 (68.9)	207 (73.1)	188 (66.4)	189 (67.3)	
≥ 2	263 (31.1)	76 (26.9)	95 (33.6)	92 (32.7)	
Pre-pregnancy BMI, kg/m^2^	21.02 (19.33, 23.05)	20.50 (19.05, 22.41)	20.77 (19.11, 22.86)	21.76 (19.85, 23.79)	< 0.001
SBP, mmHg	107.00 (99.00, 118.00)	108.00 (99.00, 118.00)	106.00 (99.00, 118.00)	108.00 (100.00, 118.00)	< 0.001
DBP, mmHg	68.00 (62.00, 73.00)	68.00 (62.00, 73.00)	67.00 (61.00, 72.00)	68.00 (62.00, 73.00)	< 0.001
WBC, x10^9^/L	9.20 (8.10, 10.60)	9.30 (8.00, 10.50)	9.00 (7.80, 10.40)	9.40 (8.30, 10.90)	0.065
Hemoglobin, g/L	115.00 (110.00, 120.00)	113.00 (109.00, 119.00)	114.00 (110.00, 121.00)	116.00 (110.00, 121.00)	0.054
Platelet count, x10^9^/L	213.00 (180.00, 240.00)	212.00 (182.00, 240.00)	211.00 (182.00, 235.00)	215.00 (176.50, 241.00)	0.958
Triglycerides, mmol/L	2.12 (1.72, 2.70)	2.00 (1.63, 2.41)	2.17 (1.72, 2.71)	2.28 (1.81, 2.91)	0.002
Total cholesterol, mmol/L	6.25 (5.56, 6.95)	6.33 (5.60, 6.96)	6.18 (5.52, 6.86)	6.23 (5.65, 7.04)	0.827
LDL-C, mmol/L	3.45 (3.03, 3.94)	3.48 (3.06, 3.99)	3.40 (2.97, 3.90)	3.44 (3.06, 3.94)	0.858
HDL-C, mmol/L	2.12 (1.89, 2.37)	2.15 (1.91, 2.42)	2.11 (1.89, 2.33)	2.10 (1.88, 2.35)	0.343
ALT, U/L	14.00 (10.00, 21.00)	16.00 (10.00, 24.00)	14.00 (10.00, 20.00)	14.00 (10.00, 20.00)	0.025
AST, U/L	18.00 (15.00, 22.00)	19.00 (16.00, 23.00)	18.00 (15.00, 22.00)	18.00 (15.00, 21.00)	0.086
Total bilirubin, µmol/L	6.60 (5.70, 8.00)	6.60 (5.60, 8.00)	6.80 (5.80, 8.20)	6.50 (5.65, 7.75)	0.198
Albumin, g/L	36.00 (34.80, 37.20)	36.10 (34.80, 37.10)	35.90 (34.50, 37.20)	36.20 (35.00, 37.45)	0.506
BUN, mmol/L	2.81 (2.41, 3.28)	2.69 (2.32, 3.09)	2.82 (2.43, 3.28)	2.90 (2.51, 3.42)	0.001
Creatinine, μmol/L	45.00 (41.00, 49.00)	43.00 (40.00, 47.00)	45.00 (41.00, 49.00)	46.00 (42.00, 50.00)	0.554
FPG, mmol/L	4.32 (4.07, 4.53)	4.26 (4.02, 4.53)	4.32 (4.05, 4.50)	4.34 (4.10, 4.61)	0.001
HbA1c, %	5.20 (5.00, 5.40)	5.20 (5.00, 5.40)	5.20 (5.00, 5.40)	5.30 (5.00, 5.50)	< 0.001
Fibrinogen, g/L	3.93 (3.56, 4.39)	3.94 (3.56, 4.35)	3.83 (3.52, 4.25)	4.05 (3.61, 4.52)	< 0.001
GDM, n (%)	179 (21.1)	33 (11.7)	61 (21.6)	85 (30.2)	< 0.001

All hematological and biochemical parameters presented in **Table 1** were measured before 24 gestational weeks during routine prenatal visits, prior to the diagnosis of GDM. T1: UA ≤ 221 µmol/L; T2: 221 µmol/L < UA ≤ 258 µmol/L; T3: UA > 258 µmol/L. BMI, body mass index; SBP, systolic blood pressure; DBP, diastolic blood pressure; WBC, white blood cell count; LDL-C, low-density lipoprotein-cholesterol; HDL-C, high-density lipoprotein-cholesterol; ALT, alanine aminotransferase; AST, aspartate aminotransferase; BUN, blood urea nitrogen; FPG, fasting plasma glucose; HbA1c, glycated hemoglobin; GDM, gestational diabetes mellitus.

### 3.2 Multivariate association between serum uric acid levels and the risk of gestational diabetes mellitus

As presented in **Table 2**, the association between maternal serum UA levels and the risk of GDM was evaluated using multivariable logistic regression models. When UA was analyzed as a continuous variable (per 1 µmol/L increase) or as a standardized variable (per 1-SD increase), a significant positive association with GDM risk was consistently observed across all models. In Model 1 (unadjusted), the ORs were 1.009 (95% CI: 1.005–1.012, P < 0.001) for continuous UA and 1.526 (95% CI: 1.297–1.796, P < 0.001) for standardized UA. Similar associations were observed in Model 2, which adjusted for age, assisted reproduction, gravidity, parity, and pre-pregnancy BMI (OR = 1.009, 95% CI: 1.005–1.012, P < 0.001; and OR = 1.519, 95% CI: 1.288–1.791, P < 0.001, respectively).

**Table 2:** Multivariate logistic regression analysis of the association between UA and GDM.

Variables	Model 1		Model 2		Model 3		Model 4	
	OR (95% CI)	P value	OR (95% CI)	P value	OR (95% CI)	P value	OR (95% CI)	P value
UA	1.009 (1.005, 1.012)	< 0.001	1.009 (1.005, 1.012)	< 0.001	1.007 (1.003, 1.010)	< 0.001	1.005 (1.001, 1.009)	0.009
Standardized UA	1.526 (1.297, 1.796)	< 0.001	1.519 (1.288, 1.791)	< 0.001	1.389 (1.170, 1.649)	< 0.001	1.274 (1.062, 1.528)	0.009
T1	Ref		Ref				Ref	
T2	2.082 (1.313, 3.299)	0.002	2.047 (1.287, 3.257)	0.002	1.920 (1.197, 3.079)	0.007	1.976 (1.203, 3.247)	0.007
T3	3.285 (2.109, 5.118)	< 0.001	3.223 (2.060, 5.042)	< 0.001	2.611 (1.643, 4.147)	< 0.001	2.468 (1.520, 4.007)	< 0.001
P for trend		< 0.001		< 0.001		< 0.001		0.001

Model 1: Unadjusted; Model 2: Adjusted for age, assisted reproduction, gravidity, parity, and pre-pregnancy BMI; Model 3: Adjusted for age, assisted reproduction, gravidity, parity, pre-pregnancy BMI, white blood cell count, hemoglobin, triglycerides, aspartate aminotransferase, blood urea nitrogen, and fibrinogen. Model 4: Adjusted for Model 3 + fasting blood glucose, and glycated hemoglobin.

UA, uric acid; GDM, gestational diabetes mellitus; BMI, body mass index; OR, odds ratio; CI, confidence interval.

To address the potential concern of overadjustment, Model 3 was constructed as a sensitivity analysis excluding fasting plasma glucose and glycated hemoglobin. In this model, the association between UA and GDM remained statistically significant and was slightly strengthened, with ORs of 1.007 (95% CI: 1.003–1.010, P < 0.001) for continuous UA and 1.389 (95% CI: 1.170–1.649, P < 0.001) for standardized UA.

In the fully adjusted Model 4, which additionally included WBC, hemoglobin, triglycerides, AST, BUN, FPG, HbA1c, and fibrinogen, the association remained statistically significant, although with a modest attenuation in effect size (OR = 1.005, 95% CI: 1.001–1.009, P = 0.009 for continuous UA; OR = 1.274, 95% CI: 1.062–1.528, P = 0.009 for standardized UA).

When UA was categorized into tertiles, compared with the lowest tertile (T1, ≤ 221 µmol/L), higher odds of GDM were consistently observed in T2 and T3 across all adjusted models. In Model 3, the ORs were 1.920 (95% CI: 1.197–3.079, P = 0.007) for T2 and 2.611 (95% CI: 1.643–4.147, P < 0.001) for T3, with a significant dose–response trend (P for trend < 0.001). In the fully adjusted Model 4, the corresponding ORs were 1.976 (95% CI: 1.203–3.247, P = 0.007) and 2.468 (95% CI: 1.520–4.007, P < 0.001), respectively (P for trend = 0.001).

### 3.3 Subgroup analysis

To identify potential effect modifiers, subgroup analyses were conducted by stratifying participants according to age, gravidity, parity, and pre-pregnancy BMI (**Table 3**). In women over 30 years of age, higher UA was significantly related to increased GDM risk, whether assessed per 1-unit increment or per 1-SD change (continuous UA: OR = 1.006; standardized UA: OR = 1.355; both P = 0.007). Comparisons by tertile showed elevated risks in T2 (OR = 2.662, P = 0.002) and T3 (OR = 3.396, P < 0.001) relative to T1. For participants with gravidity < 2, both continuous UA (OR = 1.009) and standardized UA (OR = 1.528) were positively associated with GDM risk (P = 0.002). The T3 group showed a notably elevated risk (OR = 3.638, P < 0.001). For women with gravidity ≥ 2, while continuous UA was not significantly associated with GDM, the T2 (OR = 2.032, P = 0.043) and T3 (OR = 2.138, P = 0.033) groups remained significant. Among women with parity < 2, both UA metrics were modestly but significantly associated with GDM (continuous UA: OR = 1.005; standardized UA: OR = 1.246; P = 0.048). In the tertile analysis, the T3 group showed more than twice the risk of GDM compared with T1 (OR = 2.224, P = 0.007). In contrast, associations in women with parity ≥ 2 were borderline significant for continuous (OR = 1.007, P = 0.052) and standardized UA (OR = 1.389, P = 0.052), but the T3 group still showed a significant elevation in risk (OR = 3.099, P = 0.011). Among women with a pre-pregnancy BMI below 24 kg/m^2^, each 1 µmol/L rise in UA corresponded to a 0.7% increase in GDM risk (OR = 1.007, P = 0.002), while a 1-SD increment was linked to a 39.3% higher risk (OR = 1.393, P = 0.002). Tertile analysis showed increased risk in T2 (OR = 1.877, P = 0.022) and T3 (OR = 2.606, P < 0.001). For participants with a pre-pregnancy BMI of 24 kg/m^2^ or above, GDM risk rose by 0.6% for every 1 µmol/L increase in UA (OR = 1.006, P = 0.007) and by 35.5% for each 1-SD rise (OR = 1.355, P = 0.007). In contrast, tertile-based comparisons (T2 and T3 vs. T1) showed no statistically significant differences.

**Table 3:** Multivariate subgroup analysis of the association between UA and GDM.

Subgroups	UA		Standardized UA		T2 vs T1		T3 vs T1	
	OR (95% CI)	P value	OR (95% CI) *P*	P value	OR (95% CI) *P*	P value	OR (95% CI) *P*	P value
Age								
≤ 30 years	1.002 (0.995, 1.009)	0.596	1.099 (0.776, 1.555)	0.596	1.156 (0.481, 2.779)	0.747	1.470 (0.608, 3.556)	0.392
> 30 years	1.006 (1.002, 1.011)	0.007	1.355 (1.088, 1.688)	0.007	2.662 (1.421, 4.987)	0.002	3.396 (1.847, 6.244)	< 0.001
Gravidity								
< 2	1.009 (1.003, 1.014)	0.002	1.528 (1.172, 1.992)	0.002	1.988 (0.943, 4.189)	0.071	3.638 (1.802, 7.342)	< 0.001
≥ 2	1.003 (0.998, 1.009)	0.234	1.172 (0.903, 1.520)	0.234	2.032 (1.021, 4.043)	0.043	2.138 (1.064, 4.298)	0.033
Parity								
< 2	1.005 (1.000, 1.009)	0.048	1.246 (1.002, 1.549)	0.048	1.813 (0.990, 3.318)	0.054	2.224 (1.238, 3.995)	0.007
≥ 2	1.007 (1.000, 1.014)	0.052	1.389 (0.998, 1.934)	0.052	2.246 (0.934, 5.402)	0.071	3.099 (1.294, 7.421)	0.011
Pre-pregnancy BMI								
< 24 kg/m^2^	1.007 (1.003, 1.011)	0.002	1.393 (1.129, 1.717)	0.002	1.877 (1.096, 3.214)	0.022	2.606 (1.528, 4.443)	< 0.001
≥ 24 kg/m^2^	1.006 (1.002, 1.011)	0.007	1.355 (1.088, 1.688)	0.007	4.398 (0.994, 19.468)	0.051	3.397 (0.797, 14.474)	0.098

The subgroup analysis adjusted for age, assisted reproduction, gravidity, parity, pre-pregnancy BMI, white blood cell count, hemoglobin, triglycerides, aspartate aminotransferase, blood urea nitrogen, fasting blood glucose, glycated hemoglobin, and fibrinogen.

UA, uric acid; GDM, gestational diabetes mellitus; BMI, body mass index; OR, odds ratio; CI, confidence interval.

### 3.4 Sensitivity analyses

Sensitivity analyses, based on median- and quartile-based categorizations of maternal serum UA, were performed to assess the stability of the results (**Table 4**). In the median-based approach, UA levels above 237 µmol/L were associated with a markedly greater risk of GDM compared with levels at or below this cutoff. The Model 1 indicated nearly double the risk (OR = 1.989, 95% CI: 1.416–2.794, P < 0.001), which remained significant in Model 2 (OR = 1.842, 95% CI: 1.301–2.609, P = 0.001) and persisted, though attenuated, in Model 3 (OR = 1.581, 95% CI: 1.086–2.301, P = 0.017). Based on quartile grouping of UA, and taking Q1 (≤ 213 µmol/L) as the reference, higher quartiles showed progressively greater GDM risk. In Model 3, the ORs were 2.170 (95% CI: 1.207–3.902, P = 0.010) for Q2, 2.404 (95% CI: 1.349–4.283, P = 0.003) for Q3, and 2.492 (95% CI: 1.402–4.431, P = 0.002) for Q4. A significant dose-response trend was confirmed (P for trend = 0.010).

**Table 4:** Sensitivity analysis based on median and quartile groupings of UA and its association with GDM.

Variables	Model 1		Model 2		Model 3	
	OR (95% CI)	P value	OR (95% CI)	P value	OR (95% CI)	P value
Median-based grouping						
Low UA	Ref		Ref		Ref	
High UA	1.989 (1.416, 2.794)	< 0.001	1.842 (1.301, 2.609)	0.001	1.581 (1.086, 2.301)	0.017
Quartile-based grouping						
Q1	Ref		Ref		Ref	
Q2	2.223 (1.286, 3.842)	0.004	2.263 (1.303, 3.931)	0.004	2.170 (1.207, 3.902)	0.010
Q3	2.774 (1.630, 4.721)	< 0.001	2.699 (1.578, 4.616)	< 0.001	2.404 (1.349, 4.283)	0.003
Q4	3.461 (2.045, 5.857)	< 0.001	3.478 (2.044, 5.918)	< 0.001	2.492 (1.402, 4.431)	0.002
P for trend		< 0.001		< 0.001		0.010

Model 1: Unadjusted; Model 2: Adjusted for age, assisted reproduction, gravidity, parity, and pre-pregnancy BMI; Model 3: Adjusted for age, assisted reproduction, gravidity, parity, pre-pregnancy BMI, white blood cell count, hemoglobin, triglycerides, aspartate aminotransferase, blood urea nitrogen, fasting blood glucose, glycated hemoglobin, and fibrinogen.

UA, uric acid; GDM, gestational diabetes mellitus; BMI, body mass index; OR, odds ratio; CI, confidence interval.

### 3.5 Predictive and dose–response analysis of uric acid in gestational diabetes mellitus

**Figure 1:**
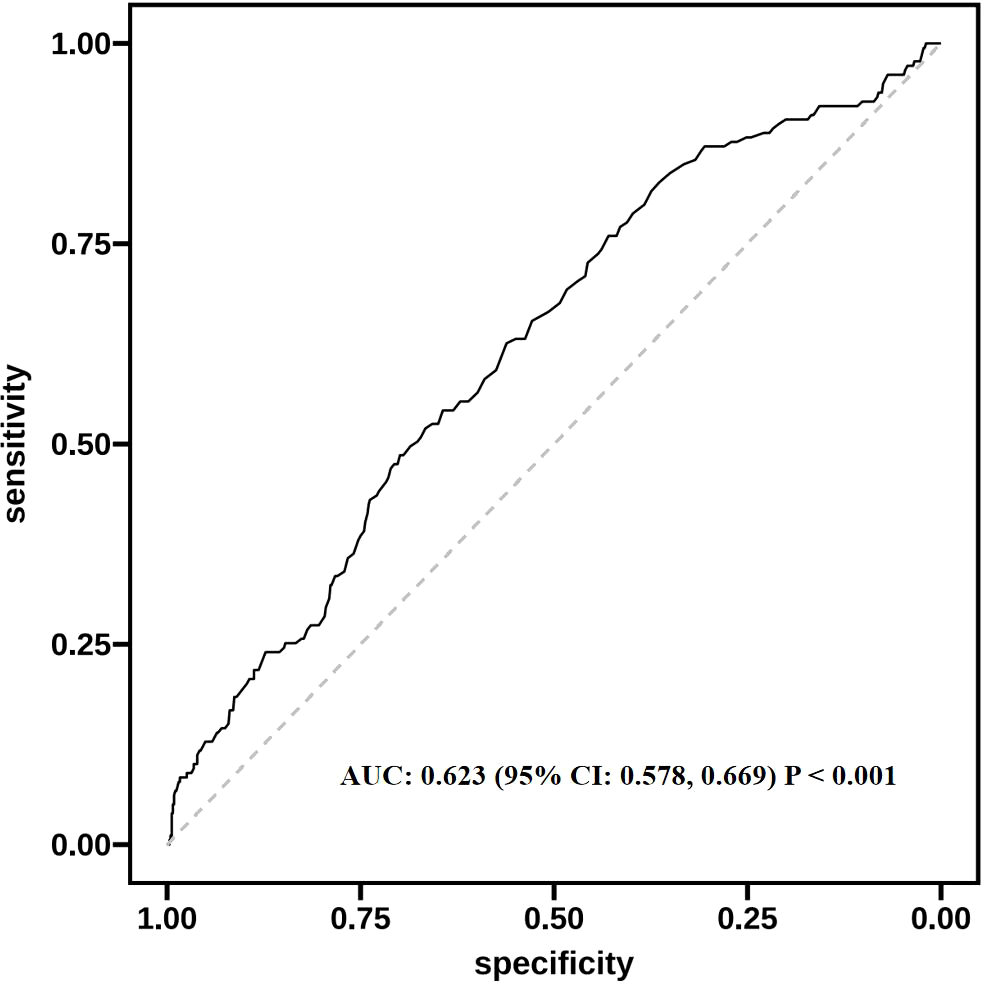
ROC curve showing the discriminatory performance of serum uric acid alone for predicting gestational diabetes mellitus. ROC, receiver operating characteristic; AUC, area under the curve; CI, confidence interval.

**Figure 2:**
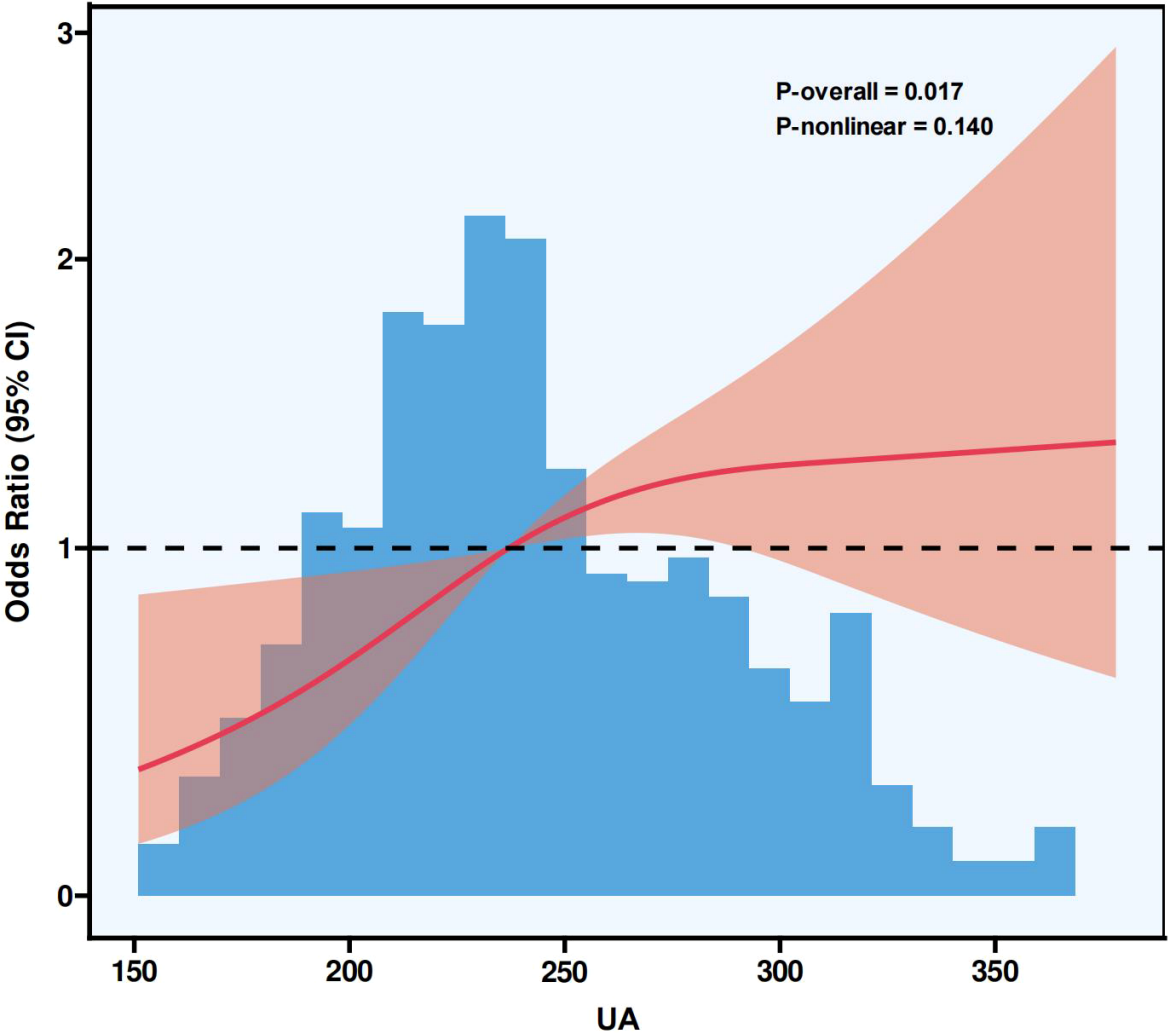
RCS plot illustrating the dose–response relationship between UA and the risk of gestational diabetes mellitus. The reference value of UA was set at the level corresponding to an odds ratio of 1.0, indicated by the horizontal dashed line. The solid line represents the estimated odds ratio, and the shaded area denotes the 95% CI. RCS, restricted cubic spline; UA, uric acid; CI, confidence interval.

ROC curve analysis (**Figure 1**) illustrated that serum UA showed a limited yet notable capacity for distinguishing GDM (AUC: 0.623, 95% CI: 0.578–0.669). After controlling for confounders, the RCS analysis in **Figure 2** showed a significant linear rise in GDM risk with increasing UA levels (P-overall = 0.017), while no nonlinear association was found (P-nonlinear = 0.140).

## 4 Discussion

This research investigates how maternal blood UA concentrations in early pregnancy relate to the likelihood of developing GDM between 24 and 28 weeks of gestation. Analysis of data from 847 pregnant participants revealed a notable positive association between higher UA levels measured before 24 weeks and elevated GDM risk. This association was consistently observed across multiple multivariable logistic regression models, including both a sensitivity analysis model excluding FPG and HbA1c, and a fully adjusted model incorporating these glycemic indicators. Given that FPG and HbA1c may lie on the potential causal pathway between UA and GDM, adjustment for these variables could introduce overadjustment. To address this concern, we constructed an additional multivariable model excluding FPG and HbA1c as a sensitivity analysis. The results showed that the positive association between early-pregnancy serum UA levels and GDM risk remained statistically significant and was slightly stronger when these glycemic variables were excluded, suggesting that adjustment for FPG and HbA1c in the fully adjusted model led to more conservative effect estimates but did not alter the overall direction or significance of the association. Furthermore, both the ROC curve and the RCS model supported the association between UA and GDM risk and indicated a dose–response relationship. However, it should be noted that the AUC value of approximately 0.623 reflects only a limited discriminatory ability. Therefore, while UA demonstrates a statistically significant association with GDM, its predictive performance as a single marker is modest. Accordingly, serum UA alone is not suitable as a standalone screening or diagnostic tool for GDM. Instead of being interpreted as a clinically actionable predictor, UA should be viewed as a risk-related biomarker that may provide incremental information for early risk stratification when considered alongside established clinical and metabolic risk factors. In this context, the clinical relevance of maternal serum UA lies primarily in its role as an indicator of an unfavorable metabolic milieu in early pregnancy, rather than as a direct guide for clinical diagnosis or intervention decisions.

It is noteworthy that several metabolic-related parameters differed significantly across UA tertile groups at baseline, suggesting a certain degree of metabolic heterogeneity among the study population. This finding indicates that higher serum UA levels may coexist with a broader dysmetabolic profile rather than representing an isolated abnormality. Importantly, because metabolic syndrome cannot be defined solely by BMI or UA concentrations, we performed extensive multivariable adjustments for key metabolic and inflammatory markers, including lipid profiles, FPG, HbA1c, liver and renal function indicators, and fibrinogen. These adjustments were intended to minimize potential confounding arising from baseline metabolic differences and to better isolate the independent association between early-pregnancy UA levels and subsequent GDM risk.

In recent years, there has been increasing focus on how maternal serum UA levels relate to the risk of GDM (18). While no universal agreement has been reached, current evidence indicates that higher UA concentrations in early or mid-pregnancy may promote insulin resistance, which could play a role in triggering GDM (8). Nevertheless, significant variation remains across different populations and regions. Such inconsistencies may stem from differences in the timing of UA measurements, research design, and analytical methods, which limit the comparability of findings. Additionally, genetic background, dietary habits, and lifestyle factors across ethnic and regional groups may also influence the observed association between UA and GDM risk. For instance, Yue and colleagues, in a retrospective study of 23,843 singleton pregnancies, found that higher maternal serum UA measured before the 24th week of pregnancy was linked to a substantially elevated risk of developing GDM (13). Specifically, when UA concentrations ranged from 240 to 300 μmol/L, the relative risk of GDM was 1.43, rising to 1.82 when the concentration exceeded 300 μmol/L. Elevated UA was also related to adverse outcomes, including preterm delivery, GDM A2, and GDM with preeclampsia, highlighting its possible value in predicting GDM onset. Similarly, in a large-scale prospective study of 85,609 pregnant participants, Zhao et al. reported a curvilinear association between UA concentrations and the risk of GDM, with a significant association only observed when UA was elevated between 13 and 18 weeks of gestation (14). Using quintile-based analysis, the relative risks of GDM for the 2nd through 5th UA quintiles were 1.11 (95% CI: 1.03–1.20), 1.27 (1.17–1.37), 1.37 (1.27–1.48), and 1.70 (1.58–1.84), respectively. This association was more pronounced among women aged 35 and above, indicating that UA testing during weeks 13–18 could offer meaningful clinical value in early GDM prevention. Furthermore, a retrospective cohort study by Pang et al., involving 18,250 mother-infant pairs, identified a notable association between higher maternal serum UA concentrations—measured at a median gestational age of 17.6 weeks—and subsequent GDM, and each SD rise in UA corresponded to a 25% greater risk of GDM (19). The study also reported a higher risk of GDM among women with hyperuricemia (OR = 1.394, P < 0.001). Beyond its link to GDM, higher UA levels have also been linked to unfavorable pregnancy outcomes, including reduced birth weight, preterm delivery, and infants small for gestational age. This indicates that even in pregnant women maintaining normal blood pressure, early-pregnancy serum UA measurements could serve as a useful indicator for identifying those at increased risk of GDM. Besides, in a cohort study of 6,000 pregnant women—comprising 1,744 GDM cases and 4,256 with normal glucose tolerance—Li et al. (20) reported that elevated serum UA in early gestation was significantly linked to a higher risk of GDM. Women in the upper UA quartiles showed greater GDM incidence, and UA levels were directly related to fasting as well as 1- and 2-hour postprandial glucose concentrations. An optimal cut-off value of 226.55 µmol/L was identified, indicating that UA may function as an independent predictor for GDM. Moreover, a systematic review and meta-analysis by Su et al. (15), incorporating 11 studies and 80,387 pregnant participants (including 9,815 with GDM), revealed elevated UA was linked to a greater risk of GDM (OR = 1.670), with the association peaking in early pregnancy (OR = 3.978 vs. 1.340 in early-to-mid gestation), indicating its potential as an early predictive marker. In contrast, Nikparast et al. analyzed data from 23 cohort studies encompassing 105,380 pregnancies and observed that women with increased UA concentrations during gestation had more than double the risk of GDM (pooled OR = 2.58) (11). The association was particularly strong before 20 weeks of gestation (OR = 3.26, 95% CI: 2.26–4.71), and subgroup analyses revealed that this relationship was more pronounced in younger pregnant women, which suggests that measuring UA during the initial stages of pregnancy could serve as an effective approach of identifying GDM risk, especially among younger populations. Additionally, Xilifu et al., through an observational study involving 684 women with GDM and 1,162 without, along with a Mendelian randomization analysis, identified a notable association between serum UA concentrations in early gestation and subsequent GDM occurrence (12). In a 1:1 matched case-control analysis, women in the highest UA tertile had nearly twice the risk of GDM relative to the lowest tertile, with risk rising further once UA surpassed 222 μmol/L. However, Mendelian randomization did not support a genetic causal association between UA and GDM (OR = 1.06, 95% CI: 0.91–1.25), suggesting that elevated UA may serve as a risk marker, rather than a direct causal factor for GDM. More recently, Liu et al. reported novel evidence from a high-risk population of Asian women undergoing assisted reproductive technology (ART), demonstrating that both pre-pregnancy serum UA levels and the UA/creatinine ratio were independently associated with the risk of GDM (21). This study extends existing evidence by focusing on women at particularly high metabolic risk and by assessing UA status prior to conception, highlighting the potential relevance of UA-related markers across different reproductive contexts and time windows. In contrast to the above studies, the present study had several notable strengths. First, this study focused on a Chinese population and assessed maternal serum UA levels measured before 24 weeks of gestation, ensuring that the exposure clearly preceded the diagnosis of GDM and strengthening the temporal validity of the observed association. Second, a comprehensive and robust statistical framework was applied, including multivariate logistic regression, sensitivity analyses using multiple categorizations of UA, and extensive subgroup analyses, which consistently supported the stability of the association across different analytical approaches. Importantly, to improve clinical interpretability, effect estimates were presented not only per 1 µmol/L increase in UA but also per 1 SD increase and across clinically meaningful categories (tertiles and quartiles), allowing a more intuitive understanding of risk associated with typical variations in UA levels. Third, RCS modeling was used to assess potential nonlinear or threshold effects and demonstrated a consistent linear dose–response relationship between early-pregnancy UA levels and GDM risk. This analysis adds value by confirming the absence of nonlinearity, thereby supporting the appropriateness of linear modeling, and strengthening the robustness of the findings. Fourth, subgroup analyses revealed that the association between UA and GDM was consistently observed across multiple clinically relevant subgroups, suggesting potential applicability across diverse maternal profiles. Taken together, these findings offer further support to existing research and expand the current understanding of the relationship between maternal serum UA and GDM. Nevertheless, given the single-center design of this study, further validation through large-scale, multicenter, and prospective investigations is still warranted to confirm the generalizability of our results.

The underlying biological mechanisms linking UA levels to GDM remain incompletely understood. However, multiple studies have proposed potential pathways from various perspectives. Current evidence suggests that UA may function not only as a passive metabolic byproduct but also as an active participant in metabolic dysregulation, particularly under conditions of increased oxidative stress and inflammation. For example, through activation of the NOD-like receptor family pyrin domain-containing 3 (NLRP3) inflammasome together with the nuclear factor kappa B (NF-κB) signaling pathway, elevated UA can induce the production of pro-inflammatory mediators such as tumor necrosis factor-alpha (TNF-α) and interleukin-6 (IL-6) (22). These inflammatory mediators are known to interfere with insulin signaling pathways by impairing insulin receptor substrate phosphorylation and downstream signaling, thereby contributing to systemic insulin resistance. The resulting oxidative stress and persistent mild inflammation subsequently worsen insulin resistance, ultimately heightening the likelihood of GDM (23). Additionally, a high-uric-acid environment may directly impair pancreatic β-cells by inducing oxidative stress and apoptosis, thereby reducing insulin synthesis and secretion, and contributing to abnormal glucose metabolism (24–26). Given the increased metabolic demands and progressive physiological insulin resistance that characterize normal pregnancy, even subtle impairments in β-cell compensatory capacity may predispose susceptible women to overt glucose intolerance and GDM. UA can also undergo reabsorption via glucose transporter 9 (GLUT-9) in the renal tubules, which elevates circulating UA levels and stimulates the renin–angiotensin–aldosterone system (RAAS), and this activation may result in sodium and fluid retention, metabolic imbalances, and aggravated insulin resistance (27, 28). Activation of RAAS has additionally been implicated in endothelial dysfunction and altered adipose tissue metabolism, which may further exacerbate insulin resistance during pregnancy. These mechanisms may act synergistically, ultimately facilitating the onset of GDM. Importantly, these pathways are not mutually exclusive and may interact within a broader network of metabolic and inflammatory disturbances characteristic of early pregnancy in women at higher metabolic risk. Nevertheless, a considerable portion of the available evidence originates from studies in non-pregnant populations or experimental animal models, and the extent to which these mechanisms operate specifically within the unique hormonal and metabolic milieu of pregnancy remains to be fully clarified. Therefore, more targeted mechanistic research in pregnant women is essential to elucidate the underlying causal pathways and to determine whether UA represents a modifiable component within the pathophysiology of GDM.

Although our findings have potential clinical relevance, a few notable limitations warrant mention. First, this was a single-center retrospective study with a limited sample size, which may introduce selection bias and limit the generalizability of the results. Second, important potential confounders, including dietary patterns, physical activity levels, socioeconomic status, and family history of diabetes, were not available due to the retrospective nature of this single-center study. These factors are known to be associated with both maternal metabolic status and the risk of GDM. The absence of these variables may have resulted in residual confounding, which could have influenced the magnitude of the observed association between early-pregnancy serum UA levels and subsequent GDM risk. Although extensive adjustment was performed for multiple available metabolic and clinical covariates, the potential impact of unmeasured lifestyle and sociodemographic factors cannot be completely excluded, and the findings should therefore be interpreted primarily as robust associations rather than definitive causal relationships. Third, serum UA levels were measured only once before 24 weeks of gestation, preventing assessment of fluctuations over the course of pregnancy. Fourth, the absence of long-term postpartum follow-up prevents us from evaluating the association between UA and future diabetes risk. Fifth, due to the retrospective design and the availability of real-world clinical data, fasting insulin or C-peptide levels were not routinely measured before 24 weeks of gestation; therefore, insulin resistance indices such as HOMA-IR or HOMA-β could not be calculated. Given that insulin resistance represents a core pathophysiological mechanism in GDM, the absence of direct insulin resistance measurements may have limited a more comprehensive mechanistic interpretation of the observed association. Sixth, multiple subgroup analyses were performed, which may increase the risk of type I error due to multiple comparisons. Although these analyses were conducted to explore the consistency and potential heterogeneity of the association across clinically relevant subgroups, the findings should be interpreted cautiously and considered exploratory in nature. Independent validation in larger, prospective studies is required to confirm these subgroup-specific observations. Finally, since the participants were limited to Chinese Han women, these findings may have limited applicability to populations of different ethnic or demographic backgrounds. Further large-scale, multicenter, prospective studies are warranted to address these limitations.

## 5 Conclusions

Overall, the study found that higher maternal UA levels early in pregnancy were linearly linked to an elevated risk of GDM. These findings indicate that elevated serum UA levels precede the onset of GDM and are associated with a higher risk of subsequent disease development, rather than serving as a definitive early diagnostic indicator.

Given the moderate predictive performance observed and the nonspecific nature of UA, our results support the potential role of UA as a risk marker or early warning signal for GDM risk stratification, rather than as a standalone diagnostic tool. Further studies should investigate the causal pathways and underlying biological mechanisms, and future well-designed prospective studies are needed to evaluate the clinical utility of UA in combination with other established metabolic and clinical markers, which may ultimately inform improved risk assessment strategies and pregnancy outcomes.

## Data Availability

The raw data supporting the conclusions of this article will be made available by the authors, without undue reservation.
